# Analysis of the effectiveness of hygiene measures and COVID-19 vaccination at a tertiary-care university hospital during the first two years of the SARS-CoV-2 pandemic

**DOI:** 10.1016/j.heliyon.2024.e30311

**Published:** 2024-04-26

**Authors:** Valentin Niekrens, Bernd Kunz, Markus Werner, Giuseppe Valenza, Christof Seggewies, Christian Bogdan, Jan Esse

**Affiliations:** a Mikrobiologisches Institut - Klinische Mikrobiologie, Immunologie und Hygiene, Universitätsklinikum Erlangen and Friedrich-Alexander-Universität (FAU) Erlangen-Nürnberg, Wasserturmstraße 3/5, D-91054, Erlangen, Germany; b Medical Informatics and Communication Center, Universitätsklinikum Erlangen, Glückstraße 11, D-91054, Erlangen, Germany; c FAU Profile Center Immunomedicine, Friedrich-Alexander-Universität (FAU) Erlangen-Nürnberg, Schlossplatz 1, D-91054, Erlangen, Germany

**Keywords:** Surveillance, Infection prevention, BNT162b2, Personal protective equipment, Hospital hygiene, Breakthrough infection

## Abstract

**Objective:**

Assessment of the effectiveness of protective measures at a tertiary-care hospital during the SARS-CoV-2 infection waves to provide advice for future pandemics.

**Design:**

Retrospective cohort study among hospital staff using in-house surveillance data.

**Setting:**

University Hospital Erlangen (UKER), a tertiary-care provider in Bavaria, Germany.

**Methods:**

We outline the preventive measures introduced at UKER and retrospectively assess their effectiveness using anonymized monitoring data that were collected during the SARS-CoV-2 pandemic from February 2020 to the end of January 2022. Analysed data includes the incidence of SARS-CoV-2 infections among employees, the frequency of high-risk contacts with infected patients or staff members and breakthrough infections considering the context of exposure.

**Results:**

The cumulative incidence of SARS-CoV-2 infections among UKER employees was higher before, but lower after the vaccination campaign when compared to the general population. Healthcare workers (HCW), notably physicians and nurses, were especially at risk of infection compared to other UKER employees with less direct patient contact (OR 1.36 [95% CI 1.18-1.57 p < 0.001]). Breakthrough infections mostly occurred after exposure during private life, i.e. in situations without protective equipment. The frequency of high-risk contacts during direct patient care remained stable after SARS-CoV-2 vaccination. Prior to vaccination, 5.2% of HCW with direct patient care tested positive for SARS-CoV-2 within 14 days. After vaccination until the onset of the Omicron wave, conversion rate dropped to 0%.

**Conclusions:**

This study provides real-world data on the effectiveness of vaccination, contact tracing, personal protective equipment and general hygiene measures during the SARS-CoV-2 pandemic. Based on our findings, we recommend a protective approach combining all these preventive measures.

## 1 Introduction

During the COVID-19 pandemic, health care workers (HCW) engaged in direct patient care were regularly exposed to severe-acute-respiratory-syndrome-coronavirus 2 (SARS-CoV-2) [1]. To ensure the safety of HCW and to maintain patient care at the University Hospital of Erlangen (UKER), a tertiary-care hospital in Bavaria, various protective measures were immediately introduced during the first wave of SARS-CoV-2 infections in Germany. These included (a) the universal wearing of protective masks by all employees; (b) sufficient air exchange in all indoor facilities; (c) a testing regimen for HCW and patients; (d) and the tracing of contacts to patients or co-workers, who had tested positive for SARS-CoV-2 based on the recommendations by the Robert Koch Institute (RKI) in Berlin, the central federal institution for disease prevention and control in Germany [2,3].

There are conflicting data on risk of HCW to become infected with SARS-CoV-2. While some studies concluded that protective measures are sufficient and infection rates are therefore not higher than in the average population [[4], [5], [6], [7]], others reported that working in direct patient care is a major risk factor for SARS-CoV-2 infection [[8], [9], [10], [11], [12]].

The development and distribution of effective SARS-CoV-2 vaccines was essential to fight the COVID-19 pandemic [13]. The SARS-CoV-2 vaccination campaign in Germany started after December 17, 2020, when the National Standing Committee on Vaccination (STIKO) published its first recommendation on COVID-19 vaccination [14]. The vaccination efforts at the UKER started on December 27, 2020. All 8560 employees of the UKER were offered the opportunity to be vaccinated by March 2021, and by May 2021 approximately 87% of the UKER employees had received a basic immunization consisting of two doses of Comirnaty®, the BNT162b2 SARS-CoV-2-mRNA vaccine of BioNTech/Pfizer [15]. After the UKER in-house vaccination campaign, UKER employees could opt for additional COVID-19 vaccinations, which were applied by the regional vaccination center or by private practitioners, making further monitoring of the vaccination rates impossible. As of April 8, 2023, 76.4% of the German population had received two vaccine doses and 62.6% have received at least one booster vaccination [16].

While the COVID-19 pandemic is currently under control, the occurrence of new viral pandemics has to be taken into consideration based on the progressing globalization [17]. Therefore, an assessment of the effectiveness of protective measures is crucial for future preparedness. In the present study, we analysed how the SARS-CoV-2 pandemic developed at UKER and whether our protective measures in combination with the vaccination campaign were sufficient to protect HCW from infection. We put a special focus on occupational high-risk contacts that occurred despite protective measures and resulted in infections among HCW.

## 2 Methods

### 2.1 Setting

**Fig. 1:**
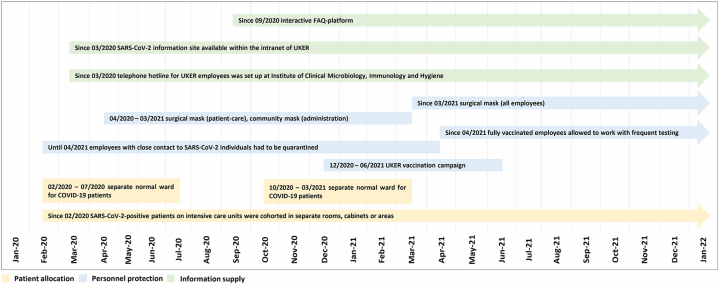
SARS-CoV-2 infection control measures implemented at UKER between 2020 and 2022 UKER, University Hospital Erlangen.

This study was based on monitoring data collected during the SARS-CoV-2 pandemic at the UKER, a tertiary-care university hospital in Bavaria, Germany with 25 clinics, 18 independent departments, 8 clinical institutes and a total of 1394 beds [18]. The study period ranged from the beginning of the pandemic in February 2020 to the end of January 2022, as reliable data on high-risk contacts and breakthrough-infections were available only until then. After this time-point, detailed contact tracing and monitoring could no longer be carried out due to rapidly increasing number of infections caused by the variant of concern (VOC) Omicron (B.1.1.529). Within the intranet of UKER, a SARS-CoV-2 information site was established that included (i) regularly updated information on diagnostic methods, general hygiene rules, the use of protective equipment, the isolation and treatment of COVID-19 patients, the isolation of contact persons and the SARS-CoV-2 testing strategy as well as (ii) an interactive FAQ-platform to simplify protective measure adherence. In addition, the Section of Hospital Hygiene within the Institute of Clinical Microbiology, Immunology and Hygiene installed a telephone hotline team, which was responsible for answering further questions on SARS-CoV-2 infection-related issues and for documentation and reporting to local health officers. An overview of the SARS-CoV-2 infection control measures implemented during the pandemic at UKER is presented in Fig. 1.

### 2.2 Patients with COVID-19

Patients with COVID-19 either were admitted to a separate normal ward for COVID-19 patients only (which was run between February 27, 2020 and July 31, 2020 and again between October 28, 2020 and March 31, 2021) or were accommodated in separate rooms, cabinets or areas within the UKER intensive care units (throughout the observation period). As far as staff availability allowed, separate nursing personnel was assigned to take care of the COVID-19 patients. To gather information about patients with SARS-CoV-2 infection at UKER, the Medical Center for Information and Communication Technology (MIK) of the UKER established the in-house web application PandIS (“pandemic information system”; v2 since April 19, 2021). The number and location of SARS-CoV-2-positive patients treated in intensive care units or on normal wards were communicated twice daily (9 a.m. and 6 p.m.) by an automated mail system. Our data were based on the daily report at 9 a.m.

### 2.3 Use of masks and personal protective equipment (PPE) at UKER

Face masks had to be worn continuously throughout the SARS-CoV-2 pandemic. All HCW, who took care of in-patients, and the patients themselves wore surgical masks. Staff without patient contact initially used community masks. From March 2021 onwards, the use of surgical facemasks as protective standard equipment became mandatory for all UKER employees, including staff without patient contact. HCW in close contact to patients tested positive for SARS-CoV-2, who could not tolerate masks or had to deposit masks due to medical reasons, routinely wore FFP-2 masks (FFP-3 masks in case of aerosol-generating procedures), safety goggles, gloves and protective gowns.

### 2.4 Hospital hygiene rules in break rooms and the staff canteen

In order to prevent high-risk contacts between employees during breaks, strict hygiene measures were defined in accordance with the recommendations of the RKI. Employees had to carry out hygienic hand disinfection before entering the room, wear their protective masks until they arrived at their seat, maintain a minimum distance of 1.5 m from other employees (e.g., ensured by markings or physical boundaries), were not allowed to exceed a maximum stay of 20 min and had to ensure air exchange by regularly ventilating the break room. In addition, it was forbidden to wear departmental clothing in the staff canteen.

### 2.5 High-risk contacts to SARS-CoV-2-positive individuals

The definition of high-risk contacts was established by the RKI and mainly focused on close proximity contacts to SARS-CoV-2-positive individuals over a certain duration without proper personal protective equipment. The RKI adjusted the details of their definitions several times [3] and the updated definitions were always adopted at UKER. From the start of the pandemic until April 14, 2021, anyone who had a high-risk contact was quarantined. Since then, fully vaccinated employees were allowed to continue working after a high-risk contact, provided they underwent regular PCR-testing [19]. The category of high-risk contacts (e.g., joint stays in break rooms, contact to index cases during work or patient care) and the participants involved (e.g., staff/staff and patient/staff) were documented in anonymized form. We used these data sets to analyse the number of high-risk contacts during direct patient care (patient/staff) in relation to the occupancy with SARS-CoV-2-positive patients measured in patient days. Furthermore, we examined how many high-risk contacts occurred during joint breaks and other working-place settings (staff/staff). A positive PCR-test result within 14 days of these contacts was recorded as contact-related infection. We defined three different observation periods according to the pandemic phases [20] in Germany as defined by the RKI (Table 1) to analyse how the ongoing changes of protective measures (e.g., introduction of vaccination) and the occurrence of SARS-CoV-2-variants affected the rate of staff/staff and patient/staff infections. Period 1 ranged from the start of the pandemic until the end of the third wave, which equals the closure of our UKER vaccination campaign on June 13, 2021. Period 2 covered the time after the completed vaccination campaign until the start of the Omicron wave on December 27, 2021. Period 3 extended from the start of the Omicron infection wave until January 31, 2022.

**Table 1:** Phases of the COVID-19 pandemic as defined by the RKI [20].

Pandemic phase	Time period in calendrical weeks	Pandemic development and predominant SARS-CoV-2 variants
Phase 0	5/2020–9/2020	First sporadic cases
Phase 1	10/2020–20/2020	First wave (ancestral virus^a^ and D614G mutant)
Phase 2	21/2020–39/2020	First summer plateau
Phase 3	40/2020–8/2021	Second wave (D614G mutant)
Phase 4	9/2021–23/2021	Third wave (VOC^b^ Alpha [B.1.1.7])
Phase 5	24/2021–30/2021	Second summer plateau
Phase 6	31/2021–51/2021	Fourth wave (VOC Delta [B.1.617.2])
Phase 7	Since 52/2021	Fifth wave (VOC Omicron [B.1.1.529])

a wild-type virus.

b variant of concern.

### 2.6 Testing strategy for HCW

HCW at the UKER had to undergo compulsory PCR testing, if they (i) developed any symptoms compatible with COVID-19, (ii) had a high-risk contact to a SARS-CoV-2-positive person, (iii) returned from a high-risk/virus variant area or country [21] or (iv) reached the end of the quarantine period after a SARS-CoV-2 infection. Swabs for PCR testing either were taken directly at the workplace (if a HCW developed symptoms at work), at home by a mobile examination team (only during phase 1 and 2 [see Table 1]) or in a testing center established outside the main UKER hospital building. HCW working on the COVID-19 wards were offered weekly voluntary PCR tests. Information about employees, who tested positive, were reported to local health authorities according to federal regulations. Reports included the likely source of infection and the vaccination status of the person. In addition, general data, such as the date of the positive SARS-CoV-2 test, the onset of symptoms and the occupational activity were collected in anonymized form. These data sets were used to evaluate the origin of breakthrough-infections, which required considering both professional (e.g., patient care) and private exposures (e.g., infected family members, infections during vacation or at private events).

Based on our vigorous testing strategy, we expect to have detected the majority of infections. In the context of an unrelated previous study we did not find anti-SARS-CoV-2 nucleocapsid antibodies in a cohort of 80 HCW without known prior infection [22], indicating that clinically asymptomatic infections were rare during the studied time period. However, due to limited sample size, a small number of undiagnosed and unreported cases cannot be excluded.

### 2.7 Statistical analyses

To determine, whether physicians and nurses were at a higher risk of SARS-CoV-2 infection than UKER employees without patient care, and to compare infection rates among HCW after high-risk contacts to patients versus contacts to employed colleagues, odds ratios (OR) with 95% confidence intervals (95% CI) were calculated using SPSS (version 28, IBM, Armonk, NY, USA). Significance of the results was assessed by chi-squared tests for dichotomous variables. The incidence of SARS-CoV-2 infections amongst UKER HCW and the general population was compared as cumulative incidence (CMI).

## 3 Results

### 3.1 Setting and participants

As of November 16, 2020, the UKER staff comprised 8560 active employees, 436 of whom had a position within the UKER administration, whereas 8124 worked in inpatient care. Of those, 2759 (32.23%) were employed as nurses and 1220 (14.25%) as physicians. The remainder of the inpatient personnel consisted of trainees, cleaning staff, housekeepers, technicians and other staff members with direct contact to patients or the hospital ward areas.

### 3.2 Incidence of SARS-CoV-2 infection amongst the UKER staff

**Fig. 2:**
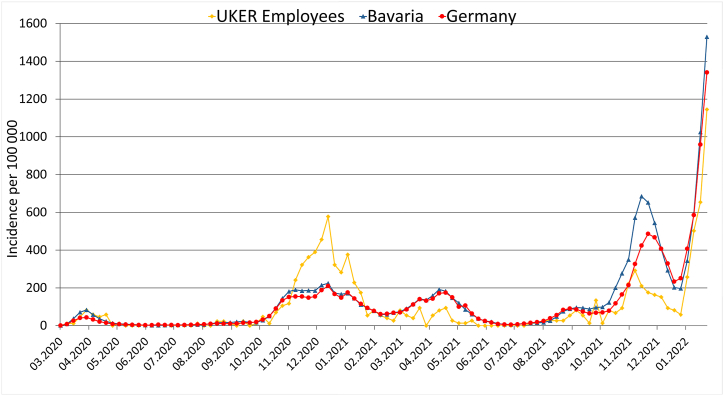
7-days SARS-CoV-2 infection incidence per 100 000 persons of the UKER employees as compared to the general population in Bavaria or Germany UKER, University Hospital Erlangen. Data for Germany and Bavaria were taken from the records of the RKI [23].

The 7-days incidence of SARS-CoV-2 infections per 100 000 persons was higher among the UKER staff than in the general population in the second wave and lower during the further course of the pandemic, especially during the third (VOC Alpha [B.1.1.7]) and fourth infection wave (VOC Delta [B.1.617.2]) after completion of the vaccination campaign (Fig. 2). This difference became even more evident by comparing the CMI amongst UKER employees and within the general population during the second (before vaccinations), third and fourth infection wave (after vaccinations) (Table 2).

**Table 2:** Cumulative incidence (CMI) of SARS-CoV-2 infections in the second, third and fourth infection wave amongst the employees of the UKER and in the general population of Bavaria and Germany.

SARS-CoV-2 infection waves	Infections at UKER (CMI)	Infections in Bavaria (CMI)	Infections in Germany (CMI)
Second Wave (40/2020–8/2021)	331 (3.87%)	369 634 (2.81%)	2 158 175 (2.60%)
Third Wave (9/2021–23/2021)	43 (0.50%)	207 360 (1.58%)	1 267 218 (1.52%)
Fourth Wave (31/2021–51/2021)	186 (2.17%)	657 941 (5.01%)	3 242 176 (3.90%)

Population size data for Bavaria and Germany were collected by the Federal Statistic Office of Germany [24]. SARS-CoV-2 case numbers for Germany and Bavaria were taken from the records of the RKI [23].

From the start of the pandemic until the end of January 2022, 847 employees tested positive for SARS-CoV-2. Of these, 160 (18.89%) were physicians and 292 (34.47%) were nurses. The remainder of the SARS-CoV-2-positive UKER personnel was working in the hospital administration (61 [7.2%]), were medical students or trainees (46 [5.43%]) or had other contacts to the hospital ward or patient areas (288 [34%]). The risk of physicians and nurses for infection was significantly higher compared to the other occupations (OR 1.36 [95% CI 1.18–1.57, p < 0.001]).

### 3.3 Breakthrough-infections

The RKI defined breakthrough-infections as symptomatic cases of SARS-CoV-2 infections that occurred at least 14 days after the completion of the COVID-19 basic immunization scheme and were confirmed by a positive PCR test [25]. By December 27, 2021, at the start of the Omicron wave, we had recorded 148 breakthrough-infections within the hospital personnel, with the first case diagnosed on March 3, 2021. Of those, 87 (58.78%) indicated a SARS-CoV-2 contact during private life, 5 (3.38%) recalled a low-risk situation at work, 21 (14.19%) could not recall any possible exposure and 35 employees did not provide information (23.65%).

### 3.4 High-risk contacts

**Fig. 3:**
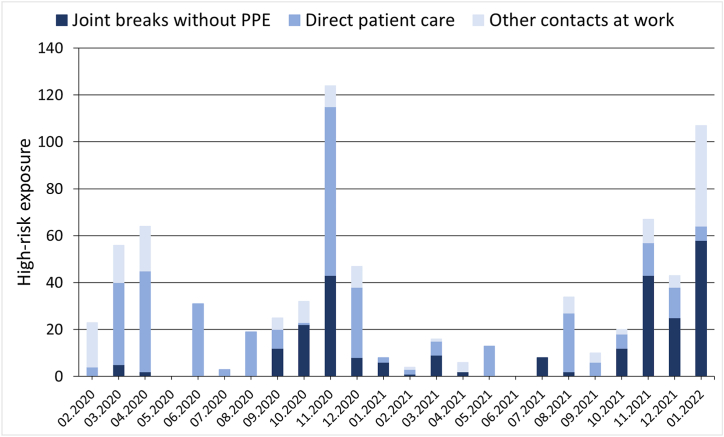
High-risk SARS-CoV-2 contacts reported by the UKER staff in absolute numbers per month PPE, personal protective equipment.

760 high-risk contacts were reported at UKER between the start of the pandemic and the end of January 2022. Of these, 339 (44.61%) occurred during direct patient care, 163 (21.54%) during other activities at UKER (e.g., office work, participation in meetings or conferences) and 258 (33.95%) during breaks together with colleagues (e.g., coffee break, lunch) (Fig. 3).

The number of high-risk contacts during direct patient care per 1000 patient-days that led to SARS-CoV-2 infections among HCW decreased after completion of the vaccination campaign and even further after the onset of the Omicron wave. The number of high-risk contacts in other UKER working places per day also dropped after vaccination, but increased again during the Omicron wave. The number of high-risk contacts per day during breaks increased both after vaccination and even further during the Omicron wave. Notably, no infections resulted from high-risk contacts in any setting after the vaccination campaign and prior to the Omicron wave (Table 3). The overall risk of infection was higher after exposure to patients than to co-workers (OR 2.74 [95% CI 1.10–6.80, p < 0.05]).

**Table 3:** Development of the absolute numbers and frequencies of occupational high-risk contacts of UKER employees and of the resulting SARS-CoV-2 infections before (period 1) and after the vaccination campaign (period 2) and during the Omicron infection wave (period 3).

Type of high-risk contact	Period 1 (05/2020-23/2021)	Period 2 (24/2021-51/2021)	Period 3 (52/2021-04/2022)
**Direct patient care**	269	63	7
Per 1000 patient days	20	15	6
Resulting Infections (rate)	14 (5.2%)	0 (0%)	1 (14.29%)
**Joint breaks without PPE**	110	80	68
Per day	0.23	0.41	1.94
Resulting infections (rate)	2 (1.82%)	0 (0%)	1 (1.47%)
**Other contacts at work**	92	27	44
Per day	0.19	0.14	1.26
Resulting infections (rate)	3 (3.26%)	0 (0%)	1 (2.27%)
**Total contacts**	471	170	119
Total infections (rate)	19 (4.03%)	0 (0%)	3 (2.52%)

PPE, personal protective equipment.

## 4 Discussion

In the present study, we assessed the effectiveness of hygiene measures to protect HCW from SARS-CoV-2 infections at UKER, a tertiary-care university hospital in Germany, during the SARS-CoV-2 pandemic. We determined the SARS-CoV-2 infection incidence of the hospital staff compared to the general population and investigated which occupational groups were particularly at risk. We payed special attention to the circumstances of high-risk contacts and examined whether they resulted in infections. In addition, the source of breakthrough-infections was analysed.

Protective measures like PPE could not prevent occasional high-risk contacts, especially during direct patient care. Presumably, it is impossible to prevent all high-risk contacts since occasional human errors will lead to gaps in safety concepts. The overall rate of SARS-CoV-2 infections following high-risk contacts was 4.03% before completion of the vaccination campaign, which is in line with previous reports [26,27]. We saw an increased risk of SARS-CoV-2 infection after contacts with patients compared to contacts with other employees (OR 2.74 [95% CI 1.10–6.80, p < 0.05]). Previous reports support a major role of direct patient contacts [28] as seen in our current analysis, whereas other studies identified contacts with co-workers and colleagues as the greater infection risk [26,29].

After completion of our institutional vaccination campaign until the start of the Omicron wave, the observed workplace-related infection rate amongst HCW at UKER dropped to 0%. This indicates that the combination of vaccination and a vigorous use of PPE is capable to prevent the vast majority of SARS-CoV-2 infections and to provide considerable safety to HCW. Nevertheless, recent findings indicate that HCW still remain at an increased risk of infection compared to the average population [1], which underlines the ongoing discussion on the impact of occupational versus non-clinical and private SARS-CoV-2 infection risks [30]. The broad definition of high-risk activities contrasts with rather low infection rates prior to vaccination and challenges the comparability of the contact situations described in different studies. Information about the index person, such as the PCR cycle-threshold-value, the time-span since symptom onset or the severity of symptoms, is usually lacking, although these factors are relevant for predicting transmissibility [31].

The differential incidence of SARS-CoV-2 infections among UKER employees compared to the general population underscores that vaccination is a crucial part of the protection concept. While prior to vaccination the incidence of infections among employees was higher, it fell below the incidence within the general population afterwards, probably because HCW were among the first to be vaccinated against COVID-19 [14]. Similar findings on the effect of HCW vaccination underline our results [32]. Our observation that the SARS-CoV-2 CMI among UKER staff remained below the incidence within the general population during the subsequent phases of the pandemic, despite rising vaccination rates in the public, indicates a good adherence to protective measures at UKER.

Breakthrough-infections mostly occurred after SARS-CoV-2 exposure during private life activities (58.78%), i.e. in situations without hygiene measures and PPE. This indicates that vaccination alone, while capable of preventing severe cases of COVID-19 [33], is not sufficient to protect against respiratory tract infections with SARS-CoV-2 in general, e.g., harmless upper respiratory tract infections. As mild infection may also lead to inability to work, vaccination of HCW alone will be insufficient to maintain patient care and to protect vulnerable patients. Therefore, education of the employees and supply of up-to date information is crucial to ensure adherence to general hygiene measures, e.g., social distancing, also in private life.

There was no increase of high-risk contacts among UKER staff with direct patient care after the vaccination campaign, measured per 1000 patient days. Therefore, HCW with direct patient contacts obviously continued to follow the hygiene rules and protective measures even after vaccination. This indicates a high level of hygiene compliance within the group of healthcare professionals. This increased level of compliance might also result from a personally perceived threat by COVID-19 and a particularly high level of responsibility for the treated patients. In contrast, there was an increase of high-risk contacts per day during common breaks of UKER employees after the vaccination. This could result from a false sense of security after vaccination, an increase in negligence during the pandemic due to exhaustion and resignation of employees, the general assumption that contacts to colleagues pose a lower risk or from an increasing fatalism. Smith et al. described fatalism for catching COVID-19 as one factor associated with non-adherence to protective measures [34]. Therefore, physical and mental fatigue of HCW poses a challenge for consequent hygiene measure adherence and asks for swift detection and countermeasures, e.g., rotation of personnel. However, a routine periodic rotation of personnel allocated to SARS-CoV-2 wards was not planned and practiced at UKER during the study period. In fact, we believe that COVID-19 patients benefitted from treatment by the best-trained staff and that the risk of SARS-CoV-2 infections and transfer to other wards was reduced by dispensing with regular staff changes. On the other hand, we cannot discount the possibility that this approach might have also caused physical and/or mental fatigue of the personnel constantly working on SARS-CoV-2 wards. However, assumptions about the attitudes of our employees are speculative and should be subject of further studies to assess possibilities for improvement in the hygiene concept. Smith et al. also identified a subjectively perceived difficulty to follow regulations as a factor associated with non-adherence. Therefore, more complex or stricter rules would not necessarily help to prevent breaches of protective measures. Overall, a good communication of hospital hygiene rules and informational penetration are essential [35].

This study has several limitations. The analysed data were primarily collected for monitoring and only secondarily used for our analysis. Consequently, from a relatively high rate of employees no information was available about likely sources of breakthrough-infections. Furthermore, we cannot formally exclude social desirability bias or recall bias in the data provided by employees. With the start of the Omicron wave, structured, anonymized documentation of the various contact parameters was no longer possible, given the rapidly increasing numbers of mostly mild infections. The increasing infection numbers in January 2022, both within the general population [23] and the UKER staff, underlines that an in-hospital protection concept only works to a limited extent if the infectivity of the pathogen increases [36], vaccine efficacy decreases [37,38] and protective measures imposed by the political decision makers are loosened and reduced [39].

A strength of our study is its real-life setting. This allowed to identify real-world effects on UKER employees over a long period of time and under changing conditions (e.g., before and after vaccination), which complements previous studies on vaccine efficacy or the protective effects of PPE under ideal circumstances. Furthermore, the structure of the UKER and its personnel were representative for a tertiary-care university hospital. Established measures during the SARS-CoV-2 pandemic closely followed the recommendations of the national health officials. Therefore, the findings of this study are presumably representative for other tertiary-care hospitals in Germany, Europe and worldwide.

Our analysis provides new real-life data on how a protective approach that combines hygiene measures and effective vaccination can help to combat a pandemic. While protecting the HCW and providing optimal safety at work, the measures also helped to maintain the functionality of the hospital and guaranteed proper patient care. Such a concept can only be successful if penetration of up-to-date information and compliance to the hygiene regulations are ensured, e.g., by interactive FAQ-tools in the hospital intranet or a telephone hotline, and high vaccination rates are achieved. Possible concerns of employees should be actively addressed in order to increase vaccination acceptance and confidence. In addition, we recommend that during a pandemic HCW are instructed to minimize exposure risks not only at work, but also during private life.

## Funding

This research received no external funding.

## Informed consent

The necessity for written informed consent was waived by the Ethics Committee, because the data were completely anonymized before analysis.

## Data availability

The original data sets of this study are available from the corresponding authors (C.B.; J.E.) upon reasonable request.

## Ethic approval

This research was approved by the Ethics Committee of the Friedrich-Alexander-University (FAU) Erlangen-Nürnberg on August 23, 2022 (registration number 22-276-Br).

## CRediT authorship contribution statement

**Valentin Niekrens:** Writing – original draft, Methodology, Formal analysis, Data curation. **Bernd Kunz:** Data curation. **Markus Werner:** Data curation. **Giuseppe Valenza:** Methodology. **Christof Seggewies:** Methodology. **Christian Bogdan:** Writing – review & editing, Supervision, Project administration, Conceptualization. **Jan Esse:** Writing – original draft, Methodology, Formal analysis, Data curation, Conceptualization.

## Declaration of competing interest

The authors declare that they have no known competing financial interests or personal relationships that could have appeared to influence the work reported in this paper.
